# Exploring Challenges of Health System in Iranian Traditional Medicine: A Qualitative Study

**DOI:** 10.4314/ejhs.v30i6.22

**Published:** 2020-11

**Authors:** Marziye Hadian, Alireza Jabbari, Hojjat Sheikhbardsiri

**Affiliations:** 1 Department of Health Care Management, Student Research Committee of School of Management and Medical Information, Isfahan University of Medical Sciences, Isfahan, Iran; 2 Health Services Management, Health Management and Economics Research Center, Isfahan University of Medical Sciences, Isfahan, Iran; 3 Social Determinants of Health Research Center, Institute for Futures Studies in Health, Kerman University of Medical Sciences, Kerman, Iran

**Keywords:** Traditional Medicine, Complementary Medicine, Substitute Medicine, Iranian Medicine, Challenge, Iran

## Abstract

**Background:**

Traditional medicine is a complete system, including diagnostic methods, etiology and treatment based on interpersonal differences.

Owing to a lack of investigations in the field of Iranian traditional medicine as well as its many present challenges, certain studies in this area can prove quite practical in identifying and solving ongoing challenges. This study investigates the challenges of the health system in Iranian traditional medicine in the context of control levers.

**Methods:**

The study was qualitative content analysis. A framework analysis, “Control Knob Approach”, was considered appropriate to promote apprehension of challenges of health systems in Iranian traditional medicine. Data were collected by purposeful sampling through in-depth and semi-structured individual interviews with 35 experts of Iranian traditional medicine. Directed content analysis was used to analyze the data, which extracted the initial codes after performing the recorded interviews on paper and immersing them in the data analysis.

**Results:**

Upon analysis of data by Iranian medicine experts, five main categories including financing, payment system, regulations, behavior and organization were defined alongside 13 subcategories.

**Conclusion:**

According to current challenges and the tendency of society to receive traditional medicine services, as well as the long history of traditional medicine in Iran, fair access to traditional medicine services should be provided. This access must be through the production of indigenous knowledge and the formulation of regulatory and educational policies and guidelines and the empowerment of relevant, healthy, effective, evidence-based and cost-effective forces.

## Introduction

Iranian traditional medicine is a complete system and school that includes diagnostic, etiological and treatment methods based on interpersonal differences (temperament) in the field of maintaining health and treatment of diseases (1–3). Traditional medicine emphasizes that health and illness are the results of a person's imbalance and balance in the whole system that surrounds him. In other words, in the treatment of diseases, instead of directly targeting the symptoms of the disease, a balance must be struck in the power of human self-healing (2).

One of the characteristics of this school: holism, belief in spiritual, physical and social identity for human beings. Emphasis on disease prevention by lifestyle modification, strengthening the spiritual aspects, the priority of food and natural therapies over drug therapies and the priority of single drugs over compound drugs and finally non-invasive treatments over invasive therapies are prominent features of traditional medicine (4).

Traditional medicine services offered by general practitioners in medical packages are based on three modes of treatment using lifestyle changes, herbal remedies and some simple therapies such as massage and cupping, etc. (5,6).

The medicines of the Iranian traditional school of medicine include the products mentioned in the authoritative sources related to this school of medicine. These drugs are made with the observance of special principles of medicinal plants and animal or mineral substances.

(4). Traces of traditional medicine can be found in almost all countries of the world (3,7). Many countries have accepted the role of traditional medicine in promoting the health and integrity of their services. In several developed countries, traditional medicine has been supported by the government in various respects and has played a good part in ensuring public health in the health system (3). Traditional Chinese medicine accounts for 40% of health care in the country (8). The insurance covers traditional treatment, medication and traditional medicine services. In Japan, China, Korea and Vietnam, and in Germany, Australia, Norway, England, Canada and the United States part of the costs are paid by insurance agencies (9,10). In recent years, the development of Iranian medicine has been emphasized by health policymakers in Iran (3). The national policies of the Supreme Leader have made a significant contribution to traditional medicine. These policies include: Recognition, explanation, promotion, development and institutionalization of traditional Iranian medicine, standardization and updating of diagnostic and therapeutic methods of traditional medicine .. The field of traditional medicine and monitoring of traditional medicine services and herbal remedies are noted (11,12). The health development program and improving the quality of life of the provinces, along with the Sixth Five-Year Development Plan, emphasize the development of traditional pharmacy and traditional medicine products, basic services of traditional Iranian medicine and the integration of approved traditional medicine services in the health system (3). At the national level, there is a growing demand for Iranian medical services. Of course, there are reports of abuses of public trust and market-makers in the Islamic and traditional scholars, which highlights the importance of ensuring public access to quality, safe and certified Iranian medicine services (13). To this end, the present study investigates the challenges of health system in Iranian traditional medicine in the context of control levers. Control knob one of the most popular models today is the use of control levers. These control knobs include issues such as financing, payment system, organization, regulation and behavior. Although each of these levers alone can affect the health system, more than one control lever is used to improve the health system, and these levers will have an indirect effect in addition to the direct effect. Due to the great importance of these levers in the decisions of the health system, in this study, they levers were used to identify challenges (14).

## Methods

**Study design**: A framework analysis, “Control Knob Approach”, was considered appropriate to promote apprehension of challenges of health systems in Iranian traditional medicine. This theory of health system control This model introduces five levers (financing, payment system, organization, rules and behavior) through which health system reforms can be implemented (15). In this method, data were gathered directly from the participants without any previous hypothesis. The study was conducted between January 2019 until November 2019. Different codes and categories were extracted using inductive approach. Subsequently, the derived codes were conceptually classified, considering their properties and dimensions.

**Setting, participants and data collection**: Study participants included 35 Iranian traditional medicine experts affiliated to Shiraz, Isfahan and Tehran universities of medical sciences. The interview questions were designed with the concurrence of the research team members and led by (AJ), and the interviews were conducted by (MH) and (HSH). Participants were chosen using a purposeful sampling method with maximum diversity. Sampling was carried out until data saturation occurred, i.e. when the researcher concluded that further interview would fail to provide new information. This qualitative study was done using in-depth and semi-structured interviews beginning with open questions, gradually continuing to more detailed ones. Interviews began with broad questions such as “Please describe your experiences of performing traditional medicine in Iran?” or “Could you explain some of the legal challenges of Iranian traditional medicine?” or “What are the obstacles for providing Iranian traditional medical services”? The interviews were taped and lasted from 25–74 minutes. The place and time of the interview were selected by agreement between the interviewer and the interviewees. Ninty percent of the interviews were held at the interviewees' workplaces while 10% of them were conducted virtually. Field notes were written during interviews to describe and interpret the responses correctly.. Also, participants were informed about the purpose of the study, the interview method, confidentiality of their information, and the right to withdraw from the study at any time.

**Data analysis**: Qualitative directed content analysis was used to analyze the data. Systematic stages were followed and simultaneous analysis was undertaken. First, recorded interviews were transcribed verbatim. Then, before coding, the transcribed text was read several times for familiarization. Codes and categories were extracted by inductive process via open coding through line-by-line reading of the text and devoting relevant codes to it. Then, categories emerged by constant comparison. Peer-check and constant comparison were used to reach consensus in coding. Data analysis was performed simultaneously and continually with the data collection procedure. Concepts were then identified upon completion and assuring the accuracy of coding.

**Reliability and validity**: The trustworthiness of the findings was determined using Lincoln and Guba' framework. According to this recommendation, four criteria of creditability, dependability, conformability, and transferability are required to ensure reliability. To increase data creditability, the researcher in this study engaged with data and the environment for 11 months while constantly making observations and compiling field notes. The dependability of data was assessed by peercheck strategies. Peer-check was performed monthly so as to ensure that the research team had a thorough discussion about the emerged data. Background information and personal interest of researchers on the corresponding topics and document maintenance were used for assessing the conformability of data. The context of the interviews, codes, and the extracted categories were reviewed by the research team and other professional colleagues in the field of qualitative research. Using sampling with maximum variation, the researchers were able to collect quite a mixed variety of different comments, observations, and interpretations.

## Results

**Demographic of participants**: The participants included 10 females and 25 males with a mean age of 42.25 ± 4.8 years, ranging from 35 to 52 years. The mean duration of working experience was 15.5 ± 3.4 years, and all participants had more than five years of experience in Iranian traditional medicine. Two of the participants were previously Vice-Chancellors for Clinical Affairs, 2 others were or had once been Vice-Chancellors for Health experience, 2 had worked as Vice-Chancellor for Food and Drug experience, 2 were Vice-Chancellor for Education, 20 were General Physicians with traditional medicine certificates, and 7 were food and drug experts with certificates for traditional medicine.

**Main results**: A total of 13 subcategories were obtained based on data analysis. After several reviewing and summarizing and based on similarities and differences, five main contributing categories were eventually formed through the content analysis methods including 1) financing, 2) payment system, 3) organization, 4) regulation and 5) behavior (Table 1).

**Table 1:** Categories and Sub-Categories of Challenges of Health Systems in Iranian Traditional Medicine

Main Theme	Categories	Sub-Categories	Codes
**Iranian Traditional Medicine** **Challenges**	Financing	-State budget - Insurance coverage	-Non-allocation of specific budget lines by the Ministry of Health -Failure to integrate Iranian medicine into the health system for funding -Undercover insurance to maximize Iranian medical services -Paying for Iranian medicine from patients' pockets
	Payment System	-Pricing -Payment Method	-Lack of maximum pricing of Iranian medical services by insurers -Ambiguity in the method of payment to traditional medicine professionals -There is no legal tariff in the private sector
	Regulations	-Monitoring -Licensing -Government Support	-Presence and prescription of drug trafficking, industrial, chemical and herbal refined powdered and blended by Atari to clients -Ambiguity in existing regulatory codes -Ambiguity in licensing and monitoring services -Inadequate supervision of Iranian medical service providers -Illegal activities of people with disabilities in the field of Iranian medicine -Invalid training certificate issued by non-qualified training centers -Lack of real and adequate supervision of the work of herbal medicine prescribers -Lack of support for drug production -Instability of policies and programs in the country
	Behavior	-Lack of Awareness -Moral Hazards - Advertising	-Lack of information and awareness on Iranian certified medical services -Weaknesses in appropriate communication within the areas involved -Deviant propaganda in the fields of traditional medicine -Create induction request from service providers -Supporting some influential -Iranian medical and complementary therapeutic interventions
	Organizing	- Scope and Depth of Service -Service provide Units -Communication	-Separation of services of Iranian medicine and modern medicine -Frequent and rapid changes in management -The shortage of Iranian medicine specialists -Shortage of Iranian medicine units in medical centers -Ambiguity in defining roles and responsibilities in service delivery -Insufficient attention to service -leveling and defining referral pathway in Iranian medical service delivery -Lack of proper training resources, service packs and service guides -Lack of quality training courses for different classes

The categories and corresponding sub-categories are described in the following sections.

**Financing**: Financing leverage means the mechanisms by which money is mobilized and relocated or equipped (to finance the activities of the health sector) as well as the mechanisms by which this money is allocated.

Regarding the achievement of this goal, all interviewees stated that the lack of allocation of a special budget line by the Ministry of Health, the payment of Iranian medicine drugs from the pockets of patients and finally the failure to insure insurance to maximize the financing of Iranian medicine services has been weakend the financing function.

**State budget**

“At present, traditional medicine services are without tariffs. That is to say, if one wishes to have a cupping or leech treatment and visits a traditional medicine physician, there is no exact and uniform tariff that all traditional medicine practitioners accept. Customary medicine now prevails, and no legal and exact tariff is the same in all offices”(Interview No .4).

“Unfortunately, the budget line for these services has not been defined yet. The public sector pricing of government services is carried out by the Ministry of Health, but there is no private pricing system” (Interview No. 2).

**Insurance coverage**

“To enter the Iranian traditional medicine to the health system so that the community can use it, first these services should be accessible to the public much cheaper and the important thing is that they and related medicines should be covered by traditional medical insurance. Now that insurers do not fall under the burden of this obligation, clients who visit specialists because of their insurance coverage and a health transformation plan, pay less than a person using traditional medicine capacity, and thus this leads to paying out of pocket at high rates in this service” (Interview No. 11).

“There is no insurance coverage for traditional medicine services currently available. Even in the health system transformation plan, it was not taken into account, considering that the payment from the pocket should have been reduced” (Interview No. 5).

**Payment system**: Payment methods in the health system are influential factors in the behavior of service providers and, of course, the quality of services. Different countries use different payment methods in their health system. Most interviewees believed that the lack of pricing of Iranian medical services by insurers (the only source of tariffs of Council is the Insurance), the uncertainty about how to pay for traditional medical services, and ultimately the lack of legal fees in the private sector, led to a weakness in payment in traditional medicine services.

## Pricing

“When there is no formal criterion for providing a medical service and no tariff is set, then anyone can provide it at any cost to the people” (Interview No. 25).

“When it is clear how much each traditional medicine service is costing to the patient and also to what extent the insurance covers their services, it makes sense to treat traditional medicine services such as the common medicine and the creation of an informal market is prevented” (Interview No. 21).

**Payment method**

“Unfortunately, there is no clear method for paying us. The exact task is not clear, and each time next year's promise is made to clarify tariffs and procedures”(Interview No. 30).

**Regulations**: Developing laws and regulations to improve people's health and enhance the functioning of the health system (access, quality, justice, accountability and cost reduction) are one of the governing tasks of the Ministry of Health.

Experts in the field of Iranian medicine stated that the existence and prescription of smuggled drugs, industrial, chemical and herbal judges powdered and combined by perfumers to customers and, etc. is one of the main challenges in the field of laws and regulations.

**Monitoring**

“One of the bad aspects is the entrance of a lot of people who don't have the necessary expertise in the field. It's very scary that all of these groceries are doing it somehow, even in the form of herbal remedies, chemical and industrial medicines, are being combined and sold to people. Some clergymen do this in the name of Islamic medicine and Shia medicine, meaning that everyone is doing it by his own belief”(Interview No. 3).

**Licensing**

“Some centers are illegally issuing certificates to these people. This has led some of these traditional medicine beneficiaries to make the benefits of this medicine such a big deal and give unauthorized and unapproved medicines to the body, which can have serious consequences for the patient, for example in most leech treatments that are under the supervision of the Ministry of Health, Swamp leeches are being used, while breeding leeches should be used”(Interview No. 14).

**Government support**

“*Well, in fact, we have the problem that there is no support for pharmaceutical products in this medicine and there is no stability and no program for it.” (Interview No. 22).*

“The rate of providing traditional medicine services in public and private hospitals in our country is “zero”, which means that there is currently no public or private hospital in our country that has allocated at least one percent of the medical services provided to patients to traditional medicine services” (Interview No. 35).

**Behavior**: The functioning of the health system and the state of health are affected in various ways by individual behavior. Changes in individual behavior can have a major impact on a person's health and the functioning of the health system.

Interviewees in this regard stated that the separation of Iranian and modern medicine services, frequent and rapid changes in management, lack of number of Iranian medicine units in medical centers, etc. are the main challenges in the field of organization.

**Lack of awareness**

“Several traditional medicine services are done in vain, such as cupping of vesicles and leech treatment by non-experts and physicians. Many of these services are useless because the person does not need the service and is misdiagnosed” (Interview No. 15).

“Most of our people think that Herbal medicines and traditional medicine services are harmless, but if these treatments are not used in the right way, they can cause serious harm” (Interview No. 26).

**Moral hazards**

“To place traditional medicine in society, we need the knowledge of the people and the companionship of modern medicine, but unfortunately we see the resistance that is more prevalent in the medical community as traditional medicine has never been a common practice in the country and because the community of physicians at any of their degrees does not receive formal education and because they were unaware of the potential of traditional medicine, so they resist it and even promote it in a bad way” (Interview No. 9).

**Advertising**

“*Unfortunately, abuse in traditional medicine has increased. Numerous illegal classes, unlicensed prescriptions. All of this endangers people's health” (Interview No. 11).*

**Organization**: The purpose of health services is to maintain or improve health. How effective the services are in this regard depends on what interventions are provided and how these services are organized. Resources should be used for interventions whose effectiveness is determined by national and local priorities.

Interviewees in this field stated that the lack of information and awareness about the approved services of Iranian medicine, the lack of proper communication within the areas involved, deviant propaganda in the field of traditional medicine, etc. are the main challenges in the field of behavior.

**Scope and depth of service**

“As you know the referral system situation in the country is not good, this is a very bad situation with traditional medicine. There is no clear and transparent system for it, as the number of specialists in this medicine is low because a physician has seven years to spend in general medicine and It takes four years for traditional medicine specialization, given the limited capacities of medical schools in this field, which may not meet the country's traditional medicine needs” (Interview No. 13).

**Service provide units**

“Honestly, since the discussion of traditional medicine, or Iranian medicine, we've had so many managerial changes that we don't even know ourselves responsible for the task. Tragedies could be a shortage of traditional medicine centers in the country” (Interview No. 23).

**Communication**

“Unfortunately, there is no effective relationship between traditional medicine and modern medicine services, and each has its criticisms of the other. In my opinion, these two together can create a better health system” (Interview No. 35).

## Discussion

The findings of the present study show that the ambiguities in the existing regulatory codes, the ambiguity in the issuance of licenses and the issuance of invalid training certificates by incompetent training centers led to inadequate supervision in traditional medicine service providers and thus the abuse of some individuals from traditional medicine. This negligence led to unlawful activities by incompetent individuals, resulting in prescribing drug trafficking, industrial, chemical, and herbal compounded powders by groceries to clients in traditional medicine that represent severe weakness in the field of traditional medicine.

Findings from the Sadeghi Weiz et al's (2017) study indicate that the intervention of unqualified specialists in the field of modern medicine is considered punishable under the Food and Drug Administration regulations: Anyone without a formal license to work in the field of dental medicine, pharmacy, physiotherapy, midwifery, physician therapy, or without a license from the Ministry of Health Medical Education, receive treatment from medical institutions or transfer their license to another or use another license, immediately, their workplace will be closed by the Ministry of Health Medical Education and sentenced to six months to three years in prison for a fine of 333$ to 3333$.

However, despite such a regulation, the legislator has not considered the issue of intervention of individuals and determining the limits of their competence in the field of traditional Iranian medicine. (2).

Also, in the study of Taghipour et al., The findings show that in the field of complementary and alternative medicine, due to the lack of sufficient scientific evidence for efficiency and effectiveness, lack of definition and appointment of authorities to handle complaints made about this medicine, medicine It has faced supplements and alternatives with challenges. The results of these studies confirm the results of the present study.(10).

Insufficient attention to service leveling and defining the referral route in providing traditional medicine services, lack of maximum pricing of traditional medicine services by insurers, lack of legal tariffs in the private sector and non-integration of traditional medicine in the health system to provide funding lead to induced demand from It is on the side of the providers, which is all a sign of poor funding and organization for this medicine.(16)

Nahin et al, (17) stated in their study titled, “Costs of complementary and alternative medicine (CAM) and frequency of visits to CAM practitioners”, that the number of visits to CAM centers in the USA has increased as a result of the provision of required financial supports (annual budgets by the national center for complementary and integrative health) as well as insurance coverage for traditional medicine services. This has, in turn, caused the introduction of this field of medicine into current medical fields, gaining special attention in current years. Studies conducted in England (18), Canada(19) and Australia (20) stipulate that most interventions in complementary medicine be covered by insurance. Traditional and complementary medicine in Iran requires national support, especially financial support from the Ministry of Health, to gain the required footing to become a well-accepted form of medicine as well as to facilitate education and research in the field of traditional medicine as a novel form of medicine (16,21). Certain procedures are also required to ensure that the high prices of traditional medicine services are covered by insurance. Studies (22,23) found that the induced demand for these services are in contrast to moral ethics and are generally encouraged by financial motivators. To reduce the severity of this problem, certain actions must be undertaken by the Health Ministry. For example, the Health Ministry must provide the knowledge and technical skills needed for traditional medicine, or insurance companies must comply to cover the costs of traditional medicine services, to control the current status.

Also, following the weakening of the budget of traditional medicine to provide services, the cost of traditional medicine is paid from the pockets of patients, which puts a heavy financial burden on them.(17).

This highlights the need for the assignment of standard tariffs by the Supreme Insurance Council. These results are consistent with the results of other studies (17,20). WHO has placed an emphasis on the welfare of families in regards to costs and expenses of health services including complementary and novel medicinal services (24). The simple reason behind this is that the patients are already suffering from the great burden of the disease itself, which must not be intensified by adding financial burdens (25). It seems that to decrease out-of-pocket pays by the patients, certain procedures must be undertaken in regards to providing insurance coverage to include expenses of traditional medicine as well.

Certain exemptions must also be made for individuals from lower strata (26). It is predicted that the high share of out-of-pocket pays for traditional medicine services will become a major challenge in the country unless certain policies are made beforehand to prevent this crisis. Such policies must be made in consideration of previous experiences and successful endeavors of the government in creating an effective system which covers the entire society (24).

Finally, weaknesses in the organization of traditional medicine services, including lack of appropriate educational resources, service packages and service guides, lack of quality education for different categories, lack of information and knowledge of approved traditional medicine services and some support lead to lack of Success in implementing traditional medicine program in the country has been (25).

In conclusion, considering existing challenges, one of the important tasks of policymakers is to create appropriate structures for the development of scientific, rational and integrated education of Iranian traditional medicine in the educational system of the country. Following the proper development of education, traditional medicine has gradually gained its prominence, preventing the presence of many specialists in the field of conflict between traditional and new medicine. Besides, the demand for induction will be greatly reduced.

Also, according to the tendency of the society to receive traditional medicine services, in line with the upstream documents and policies of the Ministry of Health, Treatment and Medical Education, should provide fair access to traditional medicine services. These services must be healthy, effective and cost effective.
